# Supplementary material for: Fluorescence Polarization Immunoassay for Determination of Enrofloxacin in Pork Liver and Chicken
Source: Molecules. 2019 Dec 5;24(24):4462. doi: 10.3390/molecules24244462 (PMC6943624; doi:10.3390/molecules24244462)
Supplement: Supplementary file 1 [file molecules-24-04462-s001.pdf]

A. AF-ENR

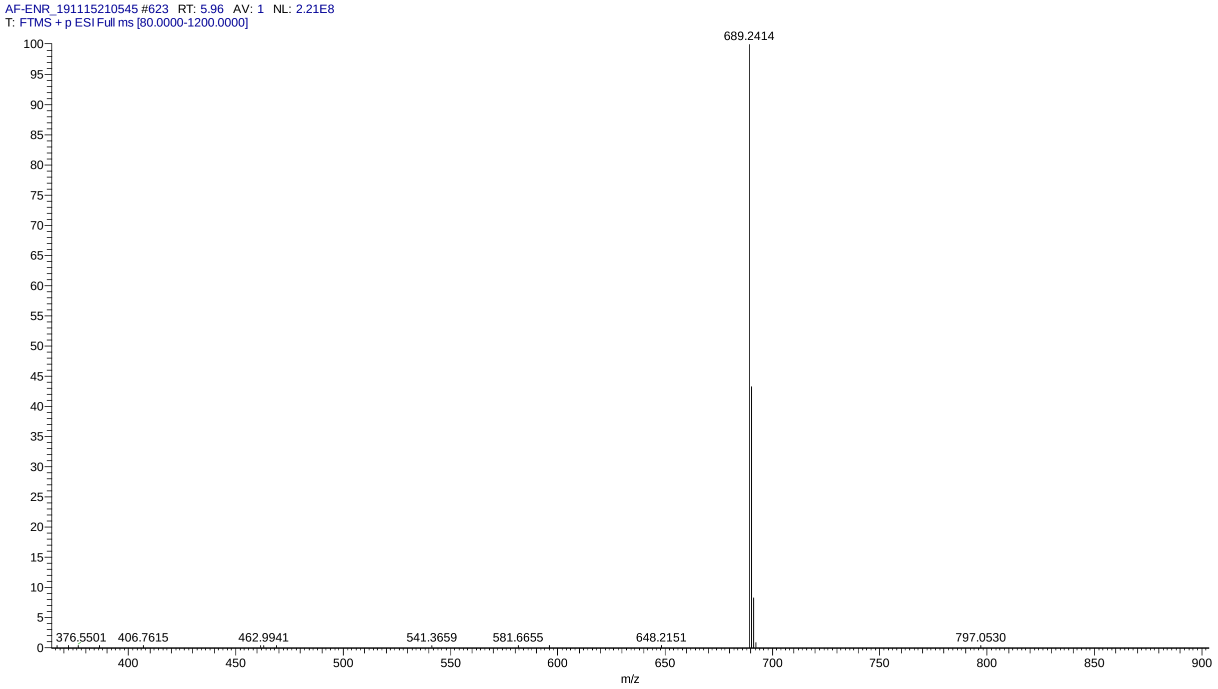

B. EDF-ENR

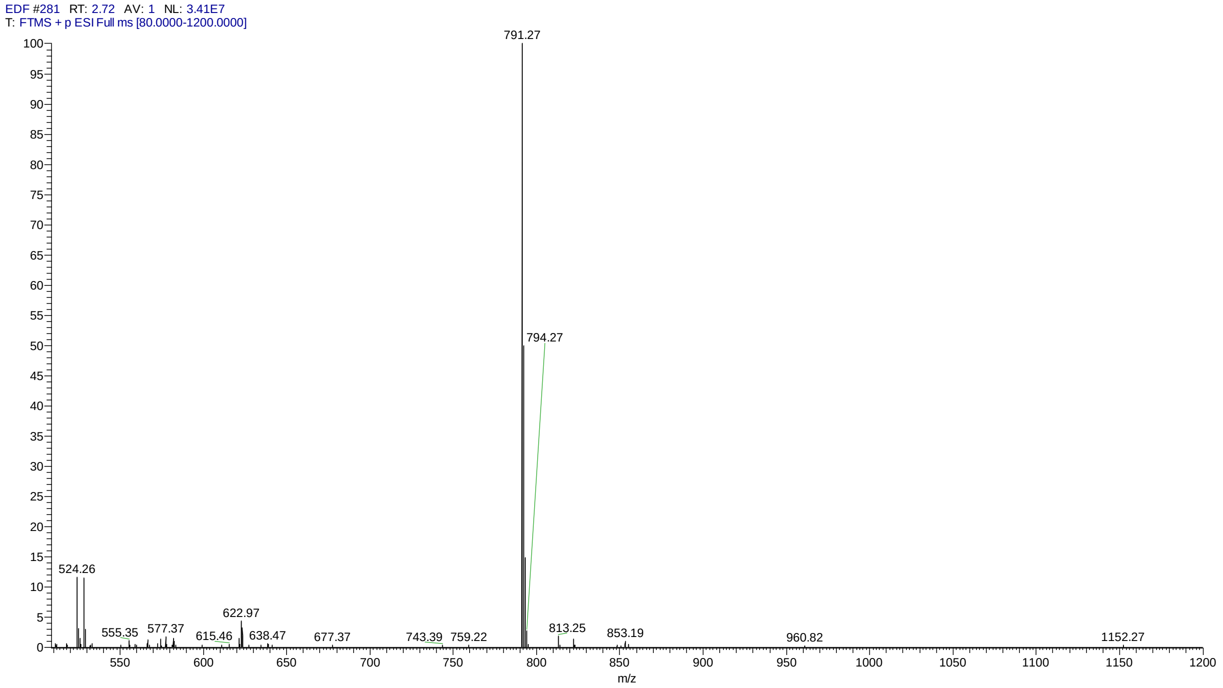

C. HDF-ENR

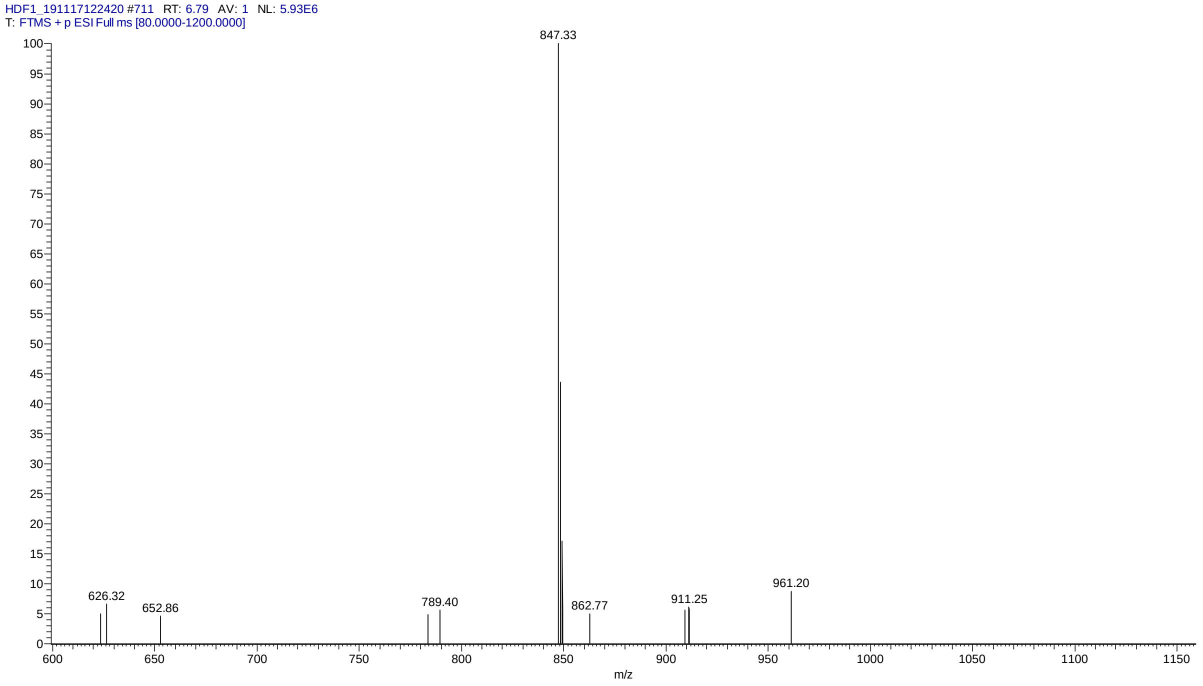

S1. Positive-ion mass spectrum (MS) of three tracers. A. AF-ENR; B. EDF-ENR; C. HDF-ENR.
